# Inflammasomes in Cancer Progression and Anti-Tumor Immunity

**DOI:** 10.3389/fcell.2022.839041

**Published:** 2022-04-20

**Authors:** Sebastian Lillo, Maya Saleh

**Affiliations:** ^1^ CNRS, ImmunoConcEpT, UMR 5164, University of Bordeaux, Bordeaux, France; > ^2^ Adjunct Professor, Department of Medicine, McGill University, Montreal, QC, Canada

**Keywords:** Inflammasome, caspase, pyroptosis, tumor, immunotherapy, macrophages, immunosuppres-sion, metastasis

## Abstract

The inflammasomes are critical regulators of innate immunity, inflammation and cell death and have emerged as important regulators of cancer development and control. Inflammasomes are assembled by pattern recognition receptors (PRR) following the sensing of microbial- or danger-associated molecular patterns (MAMPs/DAMPs) and elicit inflammation through the oligomerization and activation of inflammatory caspases. These cysteinyl-aspartate proteases cleave the proinflammatory cytokines IL-1β and IL-18 into their biologically active mature form. The roles of the inflammasomes and associated pro-inflammatory cytokines vary greatly depending on the cancer type. Here we discuss recent studies highlighting contrasting roles of the inflammasome pathway in curbing versus promoting tumorigenesis. On one hand, the inflammasomes participate in stimulating anti-tumor immunity, but they have also been shown to contribute to immunosuppression or to directly promote tumor cell survival, proliferation, and metastasis. A better understanding of inflammasome functions in different cancers is thus critical for the design of novel cancer immunotherapies.

## Introduction

Cancer development is a complex process that integrates tumor cell-intrinsic and -extrinsic signals that favor cellular transformation, unhinged growth, invasion and metastasis. Among the hallmarks of cancer (Hanahan and Weinberg, 2011), inflammation and tumor escape from immune destruction exert determining roles. Indeed, cancer control is intricately linked to the potency of the immune response. The fundamental understanding of immune surveillance, immune editing (Dunn et al., 2002) (a process in which the immune ‘pressure’ shapes tumor immunogenicity) and immune escape has resulted in breakthroughs in cancer immunotherapies, such as immune checkpoint inhibitors (ICI) or chimeric antigen receptor (CAR)-T cell therapy, among others. Such therapies have revolutionized cancer treatment by significantly improving patient survival and quality of life. Notably, the eligibility of patients to ICI immunotherapy has increased from 1.5% in 2011 to 43.6% in 2018, and >29 immunotherapies are currently approved. However, despite this impressive progress, the clinical reality reveals several unmet challenges: 1) relatively low proportions of patients exhibit objective responses to these therapies as a standalone treatment and a subset of patients develop a hyper-progressive disease, 2) immunotherapy efficacy is often associated with inflammatory toxicities that require clinical management or suspension of treatment, 3) a subset of responding patients receiving immunotherapy relapse and develop aggressive disease; 4) the efficacy of cancer therapies, including chemotherapies, ICI, adoptive T cell therapy and other immunotherapies requires signals from the microbiota; 5) systemic and cellular metabolisms impact cancer and anti-tumor immunity; and 6) immune responses promotes clonal selection, the transformation of the tumor microenvironment (TME) and the establishment of a pre-metastatic niche. The heterogeneous response of patients reflects gaps in knowledge, particularly in the understanding of organ-specific tumor macro- and micro-environments and the interactions between systems, namely that of the immune, metabolic and microbial systems in cancer control.

The innate immune system is our first line of defense, as it rapidly senses ‘danger’ signals (Matzinger, 2002) via pattern recognition receptors (PRR), e.g. Toll-like receptors (TLRs) and Nod-like receptors (NLRs) including inflammasome receptors, and provides adjuvant effects to fully engage adaptive immunity. Concerning anti-tumor immunity, PRR ligands are considered as a promising avenue in cancer immunotherapy. For instance, three TLR agonists, namely *Bacillus* Calmette-Guérin (BCG), monophosphoryl lipid A (MPL) and imiquimod are approved for use in cancer patients, and additional PRR ligands, primarily nucleic acid mimetics, are currently in clinical trial.

The inflammasomes are central effectors of innate immunity and have been demonstrated to exert important, albeit contrasting, roles in tumorigenesis, anti-tumor immunity and response to cancer therapies. In this review, we discuss recent work examining inflammasome functions in cancer and the potential of developing inflammasome-based immunotherapies.

## A Snapshot of the Inflammasomes: Structure and Activation

**FIGURE 1:**
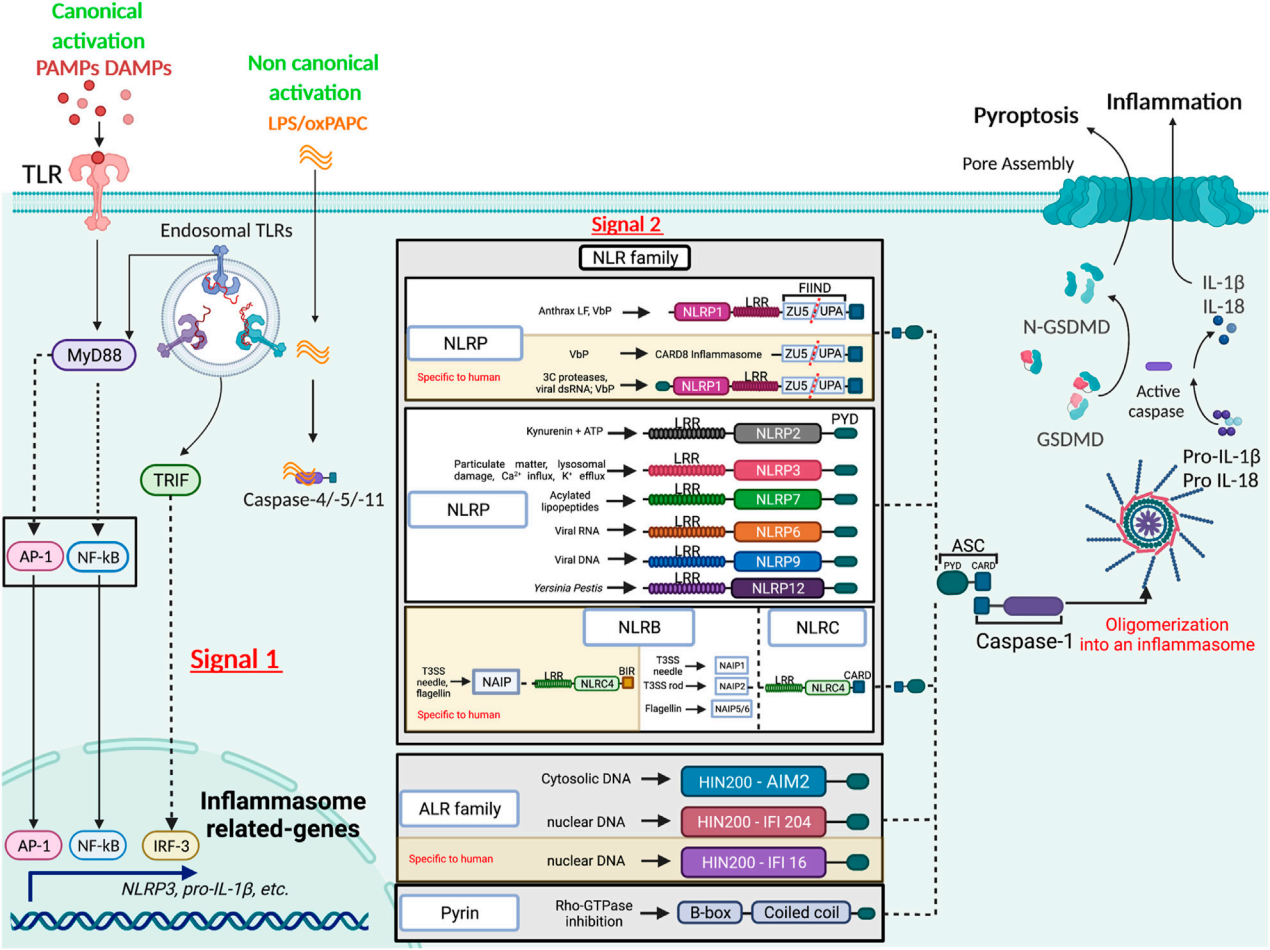
Diverse mechanisms of inflammasome activation. The activation of the inflammasome by the canonical pathway relies on two signals, the first or the priming signal allowing transcription of inflammasome components, and the second or activating signal triggering the assembly of the complex. The inflammasome scaffolding receptors belong to different families (NLR, ALR and PYRIN), each recognizing different MAMPs or DAMPs. Caspase-1 is central in catalyzing inflammasome functions, cleaving pro-inflammatory cytokines into their bioactive form and gasdermin D (GSDMD) into an N-terminal pore forming domain that elicits pyroptosis. The non-canonical route is mediated by direct binding of LPS or phospholipids to caspase-4/-5 in humans or -11 in mice.

The inflammasome term was dubbed in 2002 by Dr. Jürg Tschopp, who first described the NLRP1 (Nod-like receptor with a pyrin domain) inflammasome as an intracellular multiprotein complex, consisting of the sensor NLRP1 (Nod-like receptor with a pyrin domain), the adaptor ASC (Apoptosis-associated speck-like protein containing a CARD) and both inflammatory caspases-1 and -5 (Martinon et al., 2002). Since, more than twelve different inflammasomes have been characterized, namely NLRP1, NLRP2, NLRP3, NLRP6, NLRP7, NLRP9, NLRP12, NLCR4/NAIP, AIM2, IFI16, CARD8 and PYRIN. Most inflammasome sensors belong to the Nod-like receptor (NLR) family, which consists of 22 members in humans and 34 in mice (Ariffin and Sweet, 2013), all harboring a central nucleotide-binding and oligomerization domain (NOD) and a C-terminal leucine-rich repeat (LRR). NLR family members are further classified into four subfamilies according to the nature of their N-terminal domain, an activation domain (NLRA), a baculovirus inhibitor of apoptosis protein (IAP) repeat (BIR) domain (NLRB), a caspase activation and recruitment domain (CARD) domain (NLRC) or a pyrin domain (NLRP). Except for NLRA, members of the other NLR subfamilies have been reported to assemble inflammasomes (Figure 1). While the putative microbial or host ligands have been identified for some inflammasomes, they remain elusive for the rest. Examples of known inflammasome ligands include cytoplasmic host or pathogen (bacterial or viral) double stranded DNA that activate Absent In Melanoma (AIM)2 (Bürckstümmer et al., 2009; Fernandes-Alnemri et al., 2009; Hornung et al., 2009), nuclear viral DNA that triggers the AIM2-like receptors (ALRs) IFN-inducible factor IFI204 in mice and IFI16 in humans (Kerur et al., 2011), and intracellular bacterial proteins, namely flagellin and components of the bacterial type III secretion system such as needle and inner rod proteins, that engage NAIP/NLRC4 inflammasomes (Lightfield et al., 2008; Zhao et al., 2011; Rayamajhi et al., 2013; Yang et al., 2013; Reyes Ruiz et al., 2017; Tenthorey et al., 2017). On the other hand, the ligands of NLRP inflammasomes remain elusive. For instance, NLRP1 is activated following its proteosome-dependent “functional degradation” that liberates its C-terminal CARD to interact with caspase-1 and form an inflammasome. Such a functional degradation is induced by pathogen effectors such as *Bacillus anthracis* lethal factor (LF) (Klimpel et al., 1994; Fink et al., 2008), the protease component of lethal toxin, or *Shigella flexneri* E3 ubiquitin ligase IpaH7.8 (Sandstrom et al., 2019), and hence points to NLRP1 as a sensor of its own stability in response to pathogen activity. ATP depletion induced by infection with *Toxoplasma gondii* or *Listeria monocytogenes* also activates the NLRP1 inflammasome (Liao and Mogridge, 2013; Neiman-Zenevich et al., 2017). More recently, Bauernfried *et al.* demonstrated yet another activator of human but not mouse NLRP1, namely viral dsRNA, a bonafide ligand that binds to the leucine rich repeats (LRR) of NLRP1 (Bauernfried et al., 2021). As for NLRP1, the Pyrin inflammasome senses an “alteration” induced by infection, namely the inactivation of Rho family small GTPases by bacterial Rho-modifying toxins (Xu et al., 2014). Last, the ligands responsible for direct NLRP3 activation remain unknown. NLRP3 is activated by a wide range of MAMPs and DAMPs, including particulate matters, cholesterol crystals, cellular metabolites, lysosomal damage, defective mitophagy and ionic perturbations such as K^+^ efflux and Ca^++^ influx. NLRP3 activation also requires several post-translational modifications, i.e. ubiquitination, phosphorylation and sumoylation and the binding of NIMA-related kinase 7 (NEK7) to its LRRs (reviewed in (Swanson et al., 2019)). Activation of the non-canonical NLRP3 inflammasome is triggered by direct binding of cytosolic lipopolysaccharide (LPS) to caspase-11 in mice or caspase-4/-5 in humans (Shi et al., 2014). Oxidized phospholipid (oxPAPC) binds to caspase-11 and elicits inflammasome activation in dendritic cells (Zanoni et al., 2016) but competes with LPS binding and inhibits non-canonical inflammasome activation in macrophages conferring protection against Gram-negative bacterial sepsis (Chu et al., 2018). Last, although less well-studied, the mechanisms of activation of other NLRP inflammasomes, namely NLRP2 (Zhang et al., 2020), NLRP6 (Shen et al., 2021), NLRP7 (Radian et al., 2015), NLRP9 (reviewed in (Mullins and Chen, 2021)) and NLRP12 (reviewed in (Tuladhar and Kanneganti, 2020)) are emerging.

Inflammasome assembly leads to the multi-oligomerization of the inflammasome sensor, the ASC adaptor and the proform of caspase-1 and/or −11/−4/−5 into a perinuclear aggregate known as the ASC “speck” with prionoid features (Franklin et al., 2014). Caspase activation promotes the release of bioactive IL-1β and IL-18, and in some instances, an inflammatory cell death termed pyroptosis via the cleavage of gasdermin-D into an N-terminal domain that generates pores in the plasma membrane leading to osmotic lysis [reviewed in (Broz et al., 2020)].

The inflammasomes are best known for their function in host defence against pathogens. However, aberrant inflammasome activation has also been linked to cancer development. The role of inflammasome signaling in cancer varies according to the type of cancer, cancer etiology and cells activating the inflammasome pathway within the tumor microenvironment. In this review, we provide a synthesis of the recent literature exploring the role of different inflammasomes in cancer progression or anti-tumor immunity. We discuss evidence generated from *in vivo* mouse models, either using pharmacological inhibitors or loss-of-function genetic models, as well as evidence from patients’ cohorts and clinical trials.

## Protumoral Functions of the Inflammasome Pathway

### Promotion of Cancer Cell Survival, Proliferation and Invasion Primarily Mediated by IL-1β

**FIGURE 2:**
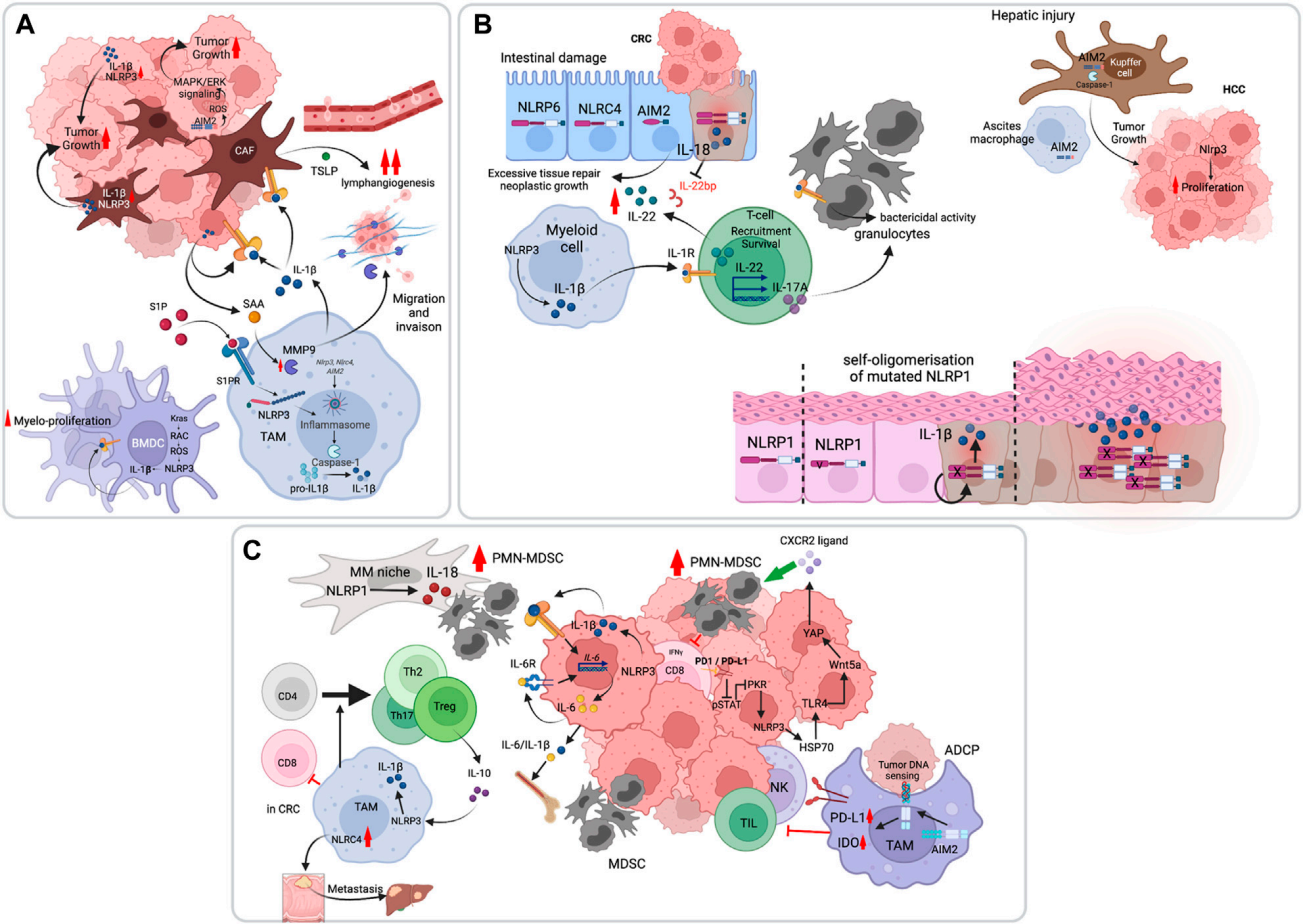
Pro-tumoral functions of the inflammasome pathway. **(A)** The inflammasome, primarily NLRP3, promotes tumor cell survival, proliferation and invasion through IL-1β. Inflammasome-independent functions of AIM2 have also been demonstrated, in which AIM2 controls mitochondrial dynamics and activate oncogenic ERK activation through reactive oxygen species (ROS). **(B)** The inflammasome can promote tumorigenesis through induction of a chronic inflammatory states. In the intestine, both IL-18 and IL-1β converge on IL-22 production which drives intestinal epithelial cell hyperproliferation. The chronic inflammatory state is also enacted by IL-17 production which recruits granulocytes to amplify the response. In the liver, hepatic injury is associated with AIM2 inflammasome activation both in Kupffer cells and in macrophages of the ascitic fluid. IL-1β in this case promotes hepatocytes hyper-proliferation. In the skin, germ-line mutations in *NLRP1* result in chronic inflammation and tumorigenesis. **(C)** The inflammasomes can indirectly favor tumor development by suppressing anti-tumor immunity. IL-1β was shown to enhance myeloid-derived suppressor cells (MDSC) recruitment to the tumor site and to promote T cell tolerogenic differentiation.

The role of the inflammasome in cancer promotion is often mediated by IL-1β, which is produced by various cells in the tumor, including tumor-associated macrophages (TAMs) and cancer-associated fibroblasts (CAFs). Indeed, myeloid- or CAF-specific deletion of genes involved in Nlrp3 inflammasome activation leads to decreased tumor growth. For example, the sphingolipid sphingosine-1-phosphate (S1P) receptor was shown to activate the Nlrp3 inflammasome in TAMs, and myeloid-specific deletion of *S1pr* reduced IL-1β-driven lymphangiogenesis and pulmonary metastasis in the polyoma middle T (PyMT) mouse model of breast cancer (Weichand et al., 2017). Using the same model, Ershaid *et al.* showed that CAF-specific genetic ablation of *Nlrp3* or *Il1b* delayed tumor growth and attenuated lung metastasis (Ershaid et al., 2019). Mechanistically, IL-1β promoted tumor progression and metastasis by driving an immunosuppressive tumor microenvironment (TME) and inducing the expression of adhesion molecules on endothelial cells (Ershaid et al., 2019). Furthermore, Nlrp3 inflammasome-mediated IL-1β was shown to induce the production of the cancer-promoting cytokine IL-22 by memory CD4^+^ T cells. This was demonstrated in models of breast and lung cancers in which neutralization of IL-1R signaling with anakinra abrogated IL-22 production and reduced tumor growth (Voigt et al., 2017). IL-1β produced by TAMs or released from cancer cells can also boost the pro-tumorigenic activity of CAFs, highlighting its role as a master orchestrator of tumorigenesis (Brunetto et al., 2019). Besides its effects on the TME, IL-1β autocrine signaling was shown to act as a direct driver of cancer cell proliferation. This was demonstrated using the diethylnitrosamine (DEN) mouse model of hepatocellular carcinoma (HCC), in which the mitophagy effector FUNDC1 was specifically deleted in hepatocytes leading to aberrant activation of the Nlrp3 inflammasome and IL-1β-driven hepatocyte hyperproliferation (Wenhui Li et al., 2019). This was also reported in a mouse model of AML driven by the oncogenic Kras^G12D^ pathway which activated the Nlrp3 inflammasome *via* Kras-RAC1-induced ROS production. *Nlrp3* deletion reduced abnormal myeloproliferation and restored normal hematopoiesis (Hamarsheh et al., 2020). In addition to inducing proliferation and survival of cancer cells, IL-1β can also favor their migration and invasion. In a colorectal cancer (CRC) model, macrophage-derived IL-1β was shown to elicit the production of serum amyloid A1 (SAA1) by tumor cells, which in a feed-forward manner induced macrophages to upregulate metalloproteinase-9 secretion allowing cancer migration and establishment of metastasis (Sudo et al., 2021) (Figure 2A). Whereas the bulk of the evidence point to IL-1β as the main driver of inflammasome tumor-promoting effects, a recent study by Hofbauer *et al.*, studying multiple myeloma in a murine model, described a role of Nlrp3-dependent IL-18 in bone destruction and multiple myeloma proliferation (Hofbauer et al., 2021).

Last, inflammasome-independent functions in tumor promotion have been reported. Qi *et al.* showed that while polydA:dT stimulation of three NSCLC cell-lines activated the AIM2 inflammasome, it did not have an impact on their growth or survival. In contrast, AIM2 promoted NSCLC tumor growth *in vivo* by regulating mitochondrial fusion dynamics and ERK activation (Qi et al., 2020).

### Promotion of Chronic Inflammation, Tissue Repair and Fibrosis

In response to tissue damage, select inflammatory cytokines promote the repair process. When this response is aberrant, as in the context of chronic inflammation, excessive tissue repair can lead to neoplastic growth. This is best illustrated in colitis-associated CRC and HCC. We and others have shown early on that inflammasome-dependent IL-18 production is necessary for intestinal tissue repair in mouse models of colitis and that excessive inflammasome signaling promoted intestinal tumorigenesis [reviewed in (Saleh and Trinchieri, 2011)]. Huber *et al.* later showed that IL-18 promoted the biological activity of IL-22, an important reparative cytokine, by inhibiting the production of IL-22-binding protein (IL-22BP), a soluble form of the IL-22 receptor and a negative regulator of IL-22 (Figure 2B). Knock-out of *Il22bp* which resulted in increased IL-22/IL-22BP ratio accelerated tumorigenesis in the dextran sulfate sodium (DSS)-azoxymethane (AOM) colitis-associated CRC mouse model as well as in *Apc* ^*min/+*^ mice that develop intestinal adenomas (Huber et al., 2012). Besides IL-18, excessive IL-1β production in the gut drives IL-17 production by CD4^+^ Th17 cells and innate lymphocytes (ILC) 3, that recruit granulocytes and trigger intestinal inflammation (Coccia et al., 2012). More recently, Dmitrieva-Posocco O *et al.* demonstrated cell-type specific function of IL-1 signaling in intestinal tumorigenesis. Whereas T cell-specific deletion of *Il1ra* reduced IL-17A/IL-22-dependent tumorigenesis, *Il1ra* ablation in neutrophils impaired their function in bacterial control, leading to microbial tumor invasion, which elicited exacerbated inflammation and CRC (Dmitrieva-Posocco et al., 2019). As in CRC, chronic inflammation and cirrhosis induced by liver injury predispose to HCC. The AIM2 inflammasome was reported to be activated in patients with advanced cirrhosis, particularly in macrophages of the ascites (Lozano-Ruiz et al., 2015) (Figure 2B). This inflammasome was later shown to drive HCC in the DEN mouse model, where *Aim2* ^*−/−*^ or *Casp1* ^*−/−*^ mice had reduced carcinogenic liver injury and cancer growth (Martínez-Cardona et al., 2018). In the human skin, NLRP1 is the predominant inflammasome sensor. In 2016, Franklin *et al.* reported gain-of-function germline mutations in *NLRP1* that lead to a familial skin inflammatory disease and associated carcinoma. The authors showed that inflammasome activation in keratinocytes leads to autocrine IL-1β that drives epidermal hyperplasia (Zhong et al., 2016).

### Promotion of Immunosuppression

Tumors use multiple means to evade anti-tumor immunity. For instance, they can usurp inflammatory pathways to establish an immunosuppressive environment. The inflammasome pathway is one such pathway demonstrated in some cancers to favor immunosuppression by promoting the genesis and recruitment of myeloid-derived suppressor cells (MDSC) or by inducing the differentiation of TAMs into a tolerogenic phenotype (Figure 2C). In 2010, using the poorly immunogenic B16-F10 melanoma model, van Deventer *et al.* showed that the Nlrp3 inflammasome suppressed the mouse response to dendritic cell tumor vaccines through MDSC recruitment (van Deventer et al., 2010). In a pancreatic ductal adenocarcinoma (PDAC) model, Daley *et al.* further implicated Nlrp3 signaling in TAM-mediated immunosuppression. They showed that deletion or inhibition of Nlrp3 inflammasome components was protective against PDAC. IL-1β mediated the tolerogenic function of Nlrp3 by regulating IL-10 production and T cell differentiation into Treg, Th2, and Th17 cells (Daley et al., 2017). Besides TAMs, PDAC tumor cells also activate the Nlrp3 inflammasome and act as a prominent source of IL-1β (Das et al., 2020). Tumor-derived IL-1β might also act *via* an IL-6-STAT3 inflammatory loop to expand MDSCs and induce immunosuppression (Tengesdal et al., 2021a). Tengesdal *et al.* have demonstrated this using the B16-F10 melanoma model and further showed that Nlrp3 inhibition with dapansutrile (OLT1177) abrogated the expression of immunosuppressives genes in PMN-MDSC and enhanced anti-tumor immunity *in vivo* (Tengesdal et al., 2021b). While NLRP3 appears to have a predominant role in dampening anti-tumor immunity, as illustrated above, other inflammasomes have also been implicated in this process. For instance, the NLRP1-IL-18 pathway was shown to promote the generation of mature MDSCs in multiple myeloma (Nakamura et al., 2018), and the Nlrc4 inflammasome in controlling CRC metastasis to the liver in non-alcoholic fatty liver disease (NAFLD) (Ohashi et al., 2019). Furthermore, the AIM2 inflammasome elicits immunosuppression following antibody-dependent cell phagocytosis (ADCP) and tumor cell DNA sensing, by upregulating PD-L1 and IDO expression, which inhibit anti-tumor immunity by NK and T cells (Su et al., 2018).

Notably, inflammasome-dependent cytokines such as IL-1β, can be produced in the TME independently of the inflammasomes. A recent work using mouse models of non-small cell lung cancer (NSCLC) and triple-negative breast cancer (TNBC) showed that IL-1β secretion by myeloid cells was independent of inflammasome activation and gasdermin D pore formation. IL-1β promoted immunosuppression by recruiting neutrophils to the tumor bed. The authors showed that tumor infiltration of neutrophils was abrogated in *Il1b* ^*−/−*^ mice, which resulted in loss of immunosuppression and restoration of anti-tumor immunity during treatment with antiangiogenic agents targeting VEGF. Although caspase-8 was activated in tumor-infiltrating myeloid cells, its deletion was not sufficient to completely block bioactive IL-1β release and neutrophil infiltration (Kiss et al., 2021). It is also worth noting that cytokine-independent functions of NLRP3 or AIM2 in immunosuppression have been documented. Petrilli and colleagues reported an immunosuppressive role of the Nlrp3-Asc-Caspase-1 pathway, that blunted NK cell tumor recruitment and activation, but that was independent of IL-1β, IL-18 or IL-1 receptor signaling. This was shown using orthotopic implantation of the breast cancer cell-line 4T1 in BALB/c mice or with MMTV-Neu^V664E^ on the BALB/c background crossed to C57Bl/6 inflammasome components-deficient mice (Guey et al., 2020). In addition, Theivanthiran *et al.* have shown that anti-PD1 immunotherapy leads to the upregulation of PD-L1 on tumor cell surface, which converges on NLRP3 activation and downstream release of heat shock protein (HSP) 70. In an autocrine and paracrine manner, HSP70 sensing by TLR4 and the subsequent expression and secretion of Wnt5a promoted the production of CXCL5 that attracts PMN-MDSCs to the tumor (Theivanthiran et al., 2021).

## Anti-Tumoral Functions of the Inflammasome Pathway

### Maintenance of Tissue Homeostasis, Promotion of Cell Differentiation and Inhibition of Proliferation

**FIGURE 3:**
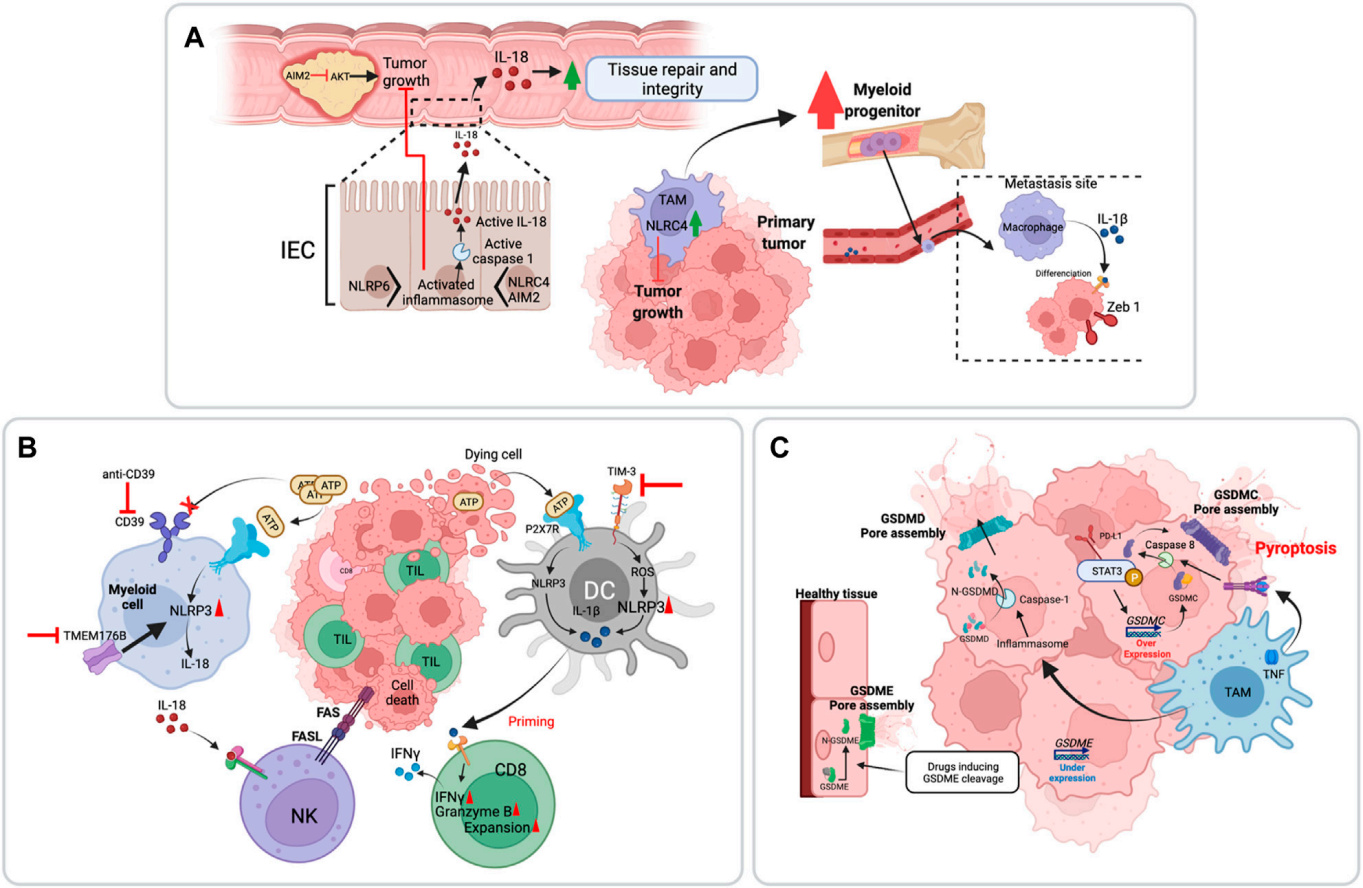
Anti-tumoral functions of the inflammasome pathway. **(A)** The inflammasome can suppress tumor cell proliferation and promotes tissue homeostasis and cell differentiation. **(B)** Inflammasome activation favors anti-tumor immunity by enhancing NK and CD8 T cell tumorilytic activities. This is primarily mediated by IL-18. Inflammasome triggers enhance the efficacy of immune checkpoint inhibitors. **(C)** Pyroptotic cell death downstream of inflammasome activation establishes an immunogenic environment through the release of several DAMPs and immunostimulatory molecules. Pyroptosis is initiated by caspase-dependent processing of gasdermins that form pores leading to osmotic lysis of the cell. Certain chemotherapies induce toxicities because of pyroptotic death of healthy cells through gasdermins E cleavage by caspase-3.

Inflammation is a protective biological response induced physiologically to counter pathogenic infection or tissue damage. Early studies by us and others have shown that the inflammasome pathway has a protective role in DSS-induced injury by promoting intestinal tissue repair via IL-18-mediated IEC regeneration (Dupaul-Chicoine et al., 2010). Concordantly, loss of Nlrp3, Asc or caspase-1 led to severe colitis but also to colitis-associated CRC (CAC) (Allen et al., 2010). Similarly, a role of Nlrp6 (Elinav et al., 2011), Nlrc4 (Rauch et al., 2017), Aim2 (Man et al., 2015) and pyrin (Sharma et al., 2018) inflammasomes in maintaining intestinal homeostasis and countering CAC was reported (Figure 3A). Flavell, Elinav and colleagues showed that this was mediated by alterations in the intestinal microbial ecology caused by lack of IL-18-instructed production of antimicrobial peptides (AMP) (Elinav et al., 2011; Levy et al., 2015). Such microbiota dysbiosis promoted neutrophil infiltration, chronic inflammation and IL-6 driven intestinal epithelial cell (IEC) hyper-proliferation (Hu et al., 2013). Besides controlling tissue homeostasis by triggering compensatory proliferation, inflammasome signalling counters tumorigenesis by upregulating differentiation programs. In the MMTV-PyMT mouse model, Castaño Z *et al*. have demonstrated an anti-metastatic role of IL-1β. They showed that myeloid-derived IL-1β imposed a differentiation “block” on metastasis-initiating cells (MICs) by upregulating ZEB1-dependent differentiation that countered their proliferative capacity (Castaño et al., 2018) (Figure 3A).

While in most instances, the underlying inflammasome mechanisms involve IL-18 or IL-1β, inflammasome-independent roles have also been described. For instance, Aim2 has been shown to restrict CAC and sporadic colon cancer through suppression of Akt signalling (Wilson et al., 2015) (Figure 3A). Inflammasome-independent functions were also reported for Nlrc4 in a subcutaneous B16-F10 melanoma mouse model. Nlrc4 expression in TAMs promoted a protective anti-tumoral response, contrary to caspase-1 or Asc, suggesting that Nlrc4 acted through an alternative mechanism (Janowski et al., 2016).

### Promotion of Anti-Tumor Immunity by Inflammasome-Dependent Cytokines

Tumor development depends on the balance between the capacity of the tumor to escape immunosurveillance and antitumor immune responses. The inflammasome-dependent cytokines IL-18 and IL-1β play a central role in anti-tumor immunity. In 2009, Ghiringhelli *et al.* have first reported that ATP release from tumor cells is sensed by the P2X7 receptor on dendritic cells (DC) which activate the Nlrp3 inflammasome, triggering the release of IL-1β. Deficiency in caspase-1 or IL-1r impaired the priming of INFγ-producing CD8^+^ T cells (Ghiringhelli et al., 2009) (Figure 3B). Consistently, IL-1β conditioning allows a better response of adoptively transferred anti-tumor T-cells. Mechanistically, IL-1β promotes T-cell homing to the tumor site and improves T cell survival and activation (Lee et al., 2019). As for IL-1β, IL-18 is an essential inducer of anti-tumor immunity. On one hand, it enhances NK cell maturation and FasL-mediated lytic activity, as demonstrated in a model of CRC metastasis to liver (Dupaul-Chicoine et al., 2015) (Figure 3B). On the other, IL-18 increases the numbers of tumor-infiltrating lymphocytes (TIL), as shown in subcutaneous model of melanoma (Zhou et al., 2020). However, IL-18 has previously failed to demonstrate efficacy in cancer clinical trials. A potential cause could be the expression of IL-18BP, a negative regulator that quenches circulating IL-18. Indeed, an IL-18 variant resistant to IL-18BP was shown to elicit increased TIL frequency and function, and to expand intra-tumoral stem-like TCF1^+^ CD8^+^ T cells in mouse tumors (Zhou et al., 2020). Akin to its role in natural anti-tumor immunity, the inflammasome also mediates synthetic anti-tumor immunity driven by certain immune checkpoint inhibitors and small molecule inhibitors of ectonucleotidases. For instance, CD39 inhibition which results in extracellular ATP accumulation enhanced anti-PD-1 anti-tumoral immune responses by engaging the P2x7-Nlrp3-IL-18 pathway (Xian-Yang Li et al., 2019; Yan et al., 2020) (Figure 3B). Similarly, unleashing Nlrp3 activity through the inhibition of Transmembrane protein 176B (TMEM176B), an immunoregulatory cation channel, enhanced TILs antitumor activity in response to immune checkpoint inhibitors (Segovia et al., 2019). More recently, Kuchroo and colleagues demonstrated that inhibition of the immune checkpoint TIM-3 on migratory DCs, resulted in strong anti-tumor immunity, mediated by ROS-driven Nlrp3 inflammasome activation (Dixon et al., 2021) (Figure 3B).

### Immunogenic Cell Death of Tumor Cells by Pyroptosis

Pyroptosis is a lytic and immunogenic regulated cell death that is induced by caspase activation. The main mechanism of pyroptosis involves GSDMD processing into a pore forming domain. The GSDM family is well conserved in vertebrates and is composed of six paralogs in humans, GSDMA, GSDMB, GSDMC, GSDMD, GSDME (DFNA5) and GSDMF (DFNB59). Mice lack a GSDMB homolog, and have three GSDMDA homologs, four GSDMC homologs, GSDMD, GSDME, and GSDMF [reviewed in (Zou et al., 2021)]. Pyroptosis of tumor cells release a spectrum of molecules allowing an efficient anti-tumoral immune response and tumor regression. This was elegantly demonstrated in a biorthogonal system in mice, which revealed that pyroptosis of less than 15% of tumor cells was sufficient to clear the entire tumor graft. Such an anti-tumoral response is mediated by a competent immune system as immunodeficient mice or T-cell deficient mice were unable to induce anti-tumor immunity (Wang et al., 2020). In addition to GSDMD, GSDME can trigger pyroptosis. Its cleavage by caspase-3 downstream of TNF or chemotherapy drugs was shown to trigger pyroptosis (Wang et al., 2017). Tumor cells, but not normal cells, tend to downregulate GSDME, which partly explains the off-target toxicity of some cancer therapies (Figure 3C). Consistently, *Gsdme* ^*−/−*^ mice were protected from chemotherapy-induced tissue damage (Wang et al., 2017). In contrast to GSDME, GSDMC is upregulated in tumor cells. Through its interaction with Stat3, PD-L1 controls GSDMC transcription. TNF released by TAMs activates caspase-8, which processes GSDMC leading to pyroptosis (Hou et al., 2020).

## Clinical Trials Targeting the Inflammasome Pathway

Multiple approaches can be used to target inflammasome activity, including the inhibition of upstream signaling pathways, inhibition of inflammasome components, or cytokine neutralization (Supplementary Table S1). Cleavage of inflammasome-dependent cytokines by caspase-1 renders it an attractive therapeutic target. Thalidomide, which targets caspase-1 is an efficient anti-inflammatory and anti-angiogenic drug. Its therapeutic use has been approved for the treatment of inflammatory skin diseases and certain types of cancer. In multiple myeloma, testing of Thalidomide in combination with other therapies have reached phase III or IV clinical trials. Other caspase-1 inhibitors used in inflammatory diseases, such as Pralnacasan or VX-765 (Belnacasan) are not yet in use in cancer clinical trials. Inhibition of specific Inflammasome receptors such as NLRP3 is also an interesting approach. Cytokine release inhibitory drug (CRID)3 also known as MCC950 and Dapansutrile (OLT1177) are NLRP3 inhibitors that have shown promising results in mice (Hamarsheh and Zeiser, 2020; Tengesdal et al., 2021b; Oizumi et al., 2021), and are expected to move into clinical trials. Nevertheless, in clinical studies, cytokine blockade is the most successful approach, and IL-1β appears to be one of the most promising targets. Based on the CANTOS trial evaluating the effect of canakinumab on preventing adverse cardiac events, Novartis is leading several clinical trials targeting IL-1β in cancer, including three in phase III in NSLC. CANOPY-A (NCT03447769) is a clinical trial combining canakinumab with cisplatin in NSLC patients following surgical resection to prevent relapse. CANOPY-1 (NCT03631199) assessed canakinumab as a first-line treatment for advanced or metastatic NSCLC in combination with pembrolizumab and platinum-based doublet chemotherapy. However, it did not reach the primary endpoints of overall survival (OS) and progression-free survival (PFS). CANOPY-2 (NCT03626545) investigated the combination of canakinumab with Docetaxel in second- or third-line therapy versus Docetaxel alone in NSCLC. However, it also did not reach its first endpoint. MABp1, a Janssen monoclonal antibody targeting IL-1α, has shown clinical benefit in a phase III clinical trial (NCT02138422), increasing the median survival of patients with CRC refractory to standard therapy. A phase II clinical trial from the Mayo Clinic (NCT00635154) investigated the effect of anakinra in patients with early-stage multiple myeloma. The results indicate that IL-1Ra suppresses markers of disease progression.

Another approach under investigation is that of stimulating anti-tumor immunity by activating the NLRP3 inflammasome. A phase III clinical trial (NCT03329846) is evaluating BMS-986299, a drug developed to activate NLRP3 inflammasome, in combination with Nivolumab in patients with advanced melanoma. The use of recombinant cytokines is also a way to promote anti-tumor activity, and SB-485232, a recombinant human IL-18 has been used in seven clinicals trials, albeit without showing efficacy.

## Conclusion

The inflammasomes play double-edged roles in tumorigenesis and anti-tumor immunity. On one hand, they promote cancer growth and metastasis by instructing an immunosuppressive TME, mainly through IL-1β, and by stimulating the proliferation of tumor cells in autocrine and paracrine amplification loops. In addition, they elicit compensatory proliferation mechanisms in response to sustained tissue damage favoring tumorigenesis. On the other hand, inflammasome activation enhances the tumorilytic activity of CD8^+^ T cells and NK cells in an IL-18-dependent manner, and further promotes anti-tumor immunity through pyroptosis, an immunogenic cell death that activates antigen-presenting cells through the release of tumor antigens and adjuvants in the TME. In view of the different inflammasome components and their downstream inflammatory cytokines, several attractive inflammasome-based therapeutic targets are currently being explored. Nevertheless, much work is still needed prior to generalizing such potential immunotherapies, as the bulk of the pre-clinical work cited here requires validation in other cancer models, translational studies and early clinical trials. In addition, taken the pleiotropic roles of the inflammasomes and their cytokines, and as with other immunotherapies, particular attention is needed to potential inflammatory toxicities or deleterious immunosuppressive effects with these approaches.
