# Supplementary material for: Inflammasomes in Cancer Progression and Anti-Tumor Immunity
Source: Front Cell Dev Biol. 2022 Apr 20;10:839041. doi: 10.3389/fcell.2022.839041 (PMC9065266; doi:10.3389/fcell.2022.839041)
Supplement: Supplementary file 1 [file Table1.docx]

**TABLE 1**

| **Therapy** | **Cancer** | **Mechanism** | **Phase** | **Trial identifier** |
| --- | --- | --- | --- | --- |
| canakinumab | Non-small-cell lung cancer | Antibody targeting IL-1 β | Phase 3 | NCT03447769 |
| canakinumab | Non-small-cell lung cancer | Antibody targeting IL-1 β | Phase 3 | NCT03626545 |
| Canakinumab | Non-small-cell lung cancer | Antibody targeting IL-1 β | Phase 3 | NCT03631199 |
| MABp1 (Xilonix) | Colorectal cancer | Antibody targeting IL-1 α | Phase 3 | NCT02138422 |
| [SB-485232](https://clinicaltrials.gov/ct2/show/NCT00659178?term=Cancer&type=Intr&cond=SB-485232&draw=2&rank=1) | Epithelia ovarian cancer | Interleukin 18 | Phase 1 | NCT00659178 |
| [SB-485232](https://clinicaltrials.gov/ct2/show/NCT00659178?term=Cancer&type=Intr&cond=SB-485232&draw=2&rank=1) | [lymphodepletion for adult patients with recurrent ovarian, fallopian tube or primary peritoneal cancer](https://clinicaltrials.gov/ct2/show/NCT02277392?term=Cancer&type=Intr&cond=SB-485232&draw=2&rank=2) | Interleukin 18 | Phase 1 | NCT02277392 |
| [SB-485232](https://clinicaltrials.gov/ct2/show/NCT00659178?term=Cancer&type=Intr&cond=SB-485232&draw=2&rank=1) | [Solid Tumors](https://clinicaltrials.gov/ct2/show/NCT00085878?term=Cancer&type=Intr&cond=SB-485232&draw=2&rank=3) | Interleukin 18 | Phase 1 | NCT00085878 |
| [SB-485232](https://clinicaltrials.gov/ct2/show/NCT00659178?term=Cancer&type=Intr&cond=SB-485232&draw=2&rank=1) | [Solid Tumors or Lymphomas](https://clinicaltrials.gov/ct2/show/NCT00085904?term=Cancer&type=Intr&cond=SB-485232&draw=2&rank=4) | Interleukin 18 | Phase 1 | NCT00085904 |
| [SB-485232](https://clinicaltrials.gov/ct2/show/NCT00659178?term=Cancer&type=Intr&cond=SB-485232&draw=2&rank=1) | [Lymphoma](https://clinicaltrials.gov/ct2/show/NCT01768338?term=Cancer&type=Intr&cond=SB-485232&draw=2&rank=6) | Interleukin 18 | Phase 1 | NCT01768338 |
| [SB-485232](https://clinicaltrials.gov/ct2/show/NCT00659178?term=Cancer&type=Intr&cond=SB-485232&draw=2&rank=1) | Lymphoma, Non-Hodgkin | Interleukin 18 | Phase 1 | NCT00500058 |
| [SB-485232](https://clinicaltrials.gov/ct2/show/NCT00659178?term=Cancer&type=Intr&cond=SB-485232&draw=2&rank=1) | [Untreated Metastatic Melanoma](https://clinicaltrials.gov/ct2/show/NCT00107718?term=Cancer&type=Intr&cond=SB-485232&draw=2&rank=5) | Interleukin 18 | Phase 2 | NCT00107718 |
| GSK1070806 | Inflammatory Bowel Diseases | Antibody targeting IL-18 | Phase 1 | NCT01035645 |
| Anakinra | Metastatic breast cancer | Antagonist to IL-1 receptor | Phase 1 | NCT01802970 |
| Anakinra | Metastatic colorectal cancer | Antagonist to IL-1 receptor | Phase 2 | NCT02090101 |
| Anakinra/dexamethasone acetate | Multiple myeloma and plasma cell neoplasm | Antagonist to IL-1 receptor | Phase 2 | NCT00635154 |
| Anakinra | Metastatic Breast Cancer | Antagonist to IL-1 receptor | Phase 1 | NCT01802970 |
| Anakinra | Metastatic Colorectal Cancer | Antagonist to IL-1 receptor | Phase 2 | NCT02090101 |
| Anakinra | Malignant Neoplasm | Antagonist to IL-1 receptor | Phase 1 | NCT01624766 |
| Anakinra | Rectal Cancer | Antagonist to IL-1 receptor | Phase 1 | NCT04942626 |
| Anakinra | Tumor, Protocol Specific | Antagonist to IL-1 receptor | Phase 1 | NCT00072111 |
| Anakinra | Pancreas Cancer | Antagonist to IL-1 receptor | Phase 1 | NCT02021422 |
| Anakinra | Metastatic Breast Cancer | Antagonist to IL-1 receptor | Phase 1 | NCT01802970 |
| Anakinra | Metastatic Colorectal Cancer | Antagonist to IL-1 receptor | Phase 2 | NCT02090101 |
| Anakinra / Denosumab /  Everolimus | advanced Malignant neoplasm / metastatic malignant neoplasm / recurrent malignant neoplasm /  refractory malignant neoplasm | Antagonist to IL-1 receptor | Phase 1 | NCT01624766 |
| Anakinra | Multiple Myeloma and Plasma Cell Neoplasm | Antagonist to IL-1 receptor | Phase 2 | NCT00635154 |
| Anakinra | B-Cell Non-Hodgkin Lymphoma | Antagonist to IL-1 receptor | Phase 2 | NCT04359784 |
| Anakinra and other drugs (Nab-paclitaxel, Gemcitabine, Cisplatin) | Pancreatic adenocarcinoma | Antagonist to IL-1 receptor | Early phase 1 | NCT02550327 |
| BMS-986299 | Advanced cancer | NLRP3 agonist | Phase 1 | NCT03444753 |
| BMS-986299 | Solid Tumor | NLRP3 agonist | Early phase 1 | NCT04541108 |
| BMS-986299 | Skin cancer (advanced melanoma) | NLRP3 agonist | Phase 3 | NCT03329846 |
| Thalidomide | Lung cancer | Inhibition of Caspase 1  activation | Phase 3 | NCT00061919 |
| Thalidomide | Multiple myeloma | Inhibition of Caspase 1  activation | Phase 3 | NCT00215943 |
| Thalidomide/CPT-11 | Gastric Cancer | Inhibition of Caspase 1  activation | Phase 4 | NCT02401971 |
| Carboplatin/paclitaxel/ thalidomide/radiation therapy | Lung Cancer | Inhibition of Caspase 1  activation | Phase 3 | NCT00004859 |
| Carboplatin, etoposide & thalidomide / Carboplatin, etoposide & placebo | Lung Cancer | Inhibition of Caspase 1  activation | Phase 3 | NCT00061919 |
| Thalidomide / CPT-11 | Gastric Cancer | Inhibition of Caspase 1  activation | Phase 4 | NCT02401971 |
| Thalidomide / leuprolide acetate / goserelin /Other: Placebo | Prostate Cancer | Inhibition of Caspase 1  activation | Phase 3 | NCT00004635 |
| Chemotherapy drugs /  thalidomide and megestrol acetate /Other: optimal support treatment | Cancer, Therapy-Related | Inhibition of Caspase 1  activation | Phase 4 | NCT03777930 |
| Biological: recombinant interferon alfa / thalidomide | Kidney Cancer | Inhibition of Caspase 1  activation | Phase 3 | NCT00005966 |
| tamoxifen citrate / thalidomide /Other: laboratory biomarker  analysis | Fallopian Tube Cancer /Primary Peritoneal Cavity Cancer /  Recurrent Ovarian Epithelial  Cancer /Stage III Ovarian Epithelial Cancer /Stage IV Ovarian  Epithelial Cancer | Inhibition of Caspase 1  activation | Phase 3 | NCT00041080 |
| Two Anti-angiogenesis Drugs Endostar and Thalidomide / | Colorectal Neoplasms | Inhibition of Caspase 1  activation | Phase 3 | NCT02748772 |
| Low dose chemotherapy | Malignant Childhood Neoplasm | Inhibition of Caspase 1  activation | Phase 3 | NCT01858571 |
| Radiation: radiation therapy /  thalidomide /Procedure: quality-of-life assessment | Tumors Metastatic to Brain | Inhibition of Caspase 1  activation | Phase 3 | NCT00033254 |
| thalidomide | Hepatocellular Carcinoma /Liver Cancer | Inhibition of Caspase 1  activation | Phase 2 /Phase 3 | NCT00728078 |
| Thalidomide / Placebo for thalidomide / Palonosetron and Dexamethasone | Neoplasms | Inhibition of Caspase 1  activation | Phase 3 | NCT02203253 |
| Thalidomide / zoledronic acid | Multiple Myeloma and Plasma Cell Neoplasm | Inhibition of Caspase 1  activation | Phase 3 | NCT00432458 |
| prednisone / thalidomide | Multiple Myeloma and Plasma Cell Neoplasm | Inhibition of Caspase 1  activation | Phase 3 | NCT00049673 |
| dexamethasone / pamidronate disodium / thalidomide / zoledronic acid | Multiple Myeloma and Plasma Cell Neoplasm | Inhibition of Caspase 1  activation | Phase 3 | NCT00033332 |
| Biological: filgrastim /Biological: recombinant interferon alfa /  cyclophosphamide /  dexamethasone / doxorubicin  hydrochloride / melphalan /  thalidomide / vincristine sulfate /Procedure: bone marrow ablation with stem cell support /Procedure: peripheral blood stem cell  transplantation | Multiple Myeloma and Plasma Cell Neoplasm | Inhibition of Caspase 1  activation | Phase 3 | NCT00028886 |
| Conmana / Thalidomide | NSCLC | Inhibition of Caspase 1  activation | Phase 4 | NCT02778893 |
| Thalidomide / Ara-C / BCNU / Cisplatin / Cytoxan / Dexamethasone / Doxorubicin / Etoposide / Filgrastim / Recombinant GM-CSF / Interferon-alpha-2b / Melphalan / Vincristine | Multiple Myeloma | Inhibition of Caspase 1  activation | Phase 3 | NCT00083551 |
| Thalidomide plus dexamethasone / Dexamethasone | Multiple Myeloma | Inhibition of Caspase 1  activation | Phase 3 | NCT01296503 |
| Thalidomide | Multiple Myeloma | Inhibition of Caspase 1  activation | Phase 3 | NCT00038233 |
| thalidomide / placebo | Multiple Myeloma | Inhibition of Caspase 1  activation | Phase 3 | NCT00218855 |
| Bortezomib, Melphalan, Prednisone, Thalidomide / Bortezomib, Melphalan, Prednisone | Multiple Myeloma | Inhibition of Caspase 1  activation | Phase 3 | NCT01063179 |
| Thalidomide / placebo / Gefitinib / Aspirin | NSCLC | Inhibition of Caspase 1  activation | Phase 2 /Phase 3 | NCT02387086 |
| A (Thalidomide + Dexamethasone) / B (Placebo + Dexamethasone) | Multiple Myeloma | Inhibition of Caspase 1  activation | Phase 3 | NCT00057564 |
| Bortezomib / Thalidomide | Multiple Myeloma | Inhibition of Caspase 1  activation | Phase 4 | NCT00652041 |
| Thalidomide / melphalan, prednisone / melphalan, prednisone, thalidomide | Newly Diagnosed, Multiple Myeloma | Inhibition of Caspase 1  activation | Phase 3 | NCT00644306 |
| Bortezomib / Thalidomide | Multiple Myeloma | Inhibition of Caspase 1  activation | Phase 3 | NCT00602511 |
| Thalidomide, Cyclophosphamide, Dexamethasone / Thalidomide, Dexamethasone / Thalidomide, Melphalan, Prednisone | Multiple Myeloma | Inhibition of Caspase 1  activation | Phase 3 | NCT01532856 |
| Thalidomide, Dexamethasone / Vincristin , Adriamycin, Dexamethasone = VAD / Thalidomide,  melphalan, endoxan, dexamethasone (MCDex-Thal) /  melphalan, endoxan, dexamethasone (MCDex) | Multiple Myeloma de Novo Treatment | Inhibition of Caspase 1  activation | Phase 3 | NCT01070862 |
| Thalidomide / Melphalan+  Prednisolone | Multiple Myeloma | Inhibition of Caspase 1  activation | Phase 3 | NCT00934154 |
| Thalidomide | Multiple Myeloma | Inhibition of Caspase 1  activation | Phase 3 | NCT00232934 |
| thalidomide / interferon alpha | Multiple Myeloma | Inhibition of Caspase 1  activation | Phase 3 | NCT00633542 |
| Velcade, Thalidomide, and Dexamethasone / Velcade, Melphalan, and Dexamethasone | Multiple Myeloma | Inhibition of Caspase 1  activation | Phase 3 | NCT00573391 |
| Thalidomide / Dexamethasone | Multiple Myeloma | Inhibition of Caspase 1  activation | Phase 3 | NCT00452569 |
| Thalidomide / Dexamethasone / DOXIL | Multiple Myeloma | Inhibition of Caspase 1  activation | Phase 3 | NCT00097981 |
| VBMCP/VBAD/Velcade /  Thalidomide/Dexamethasone / Velcade/Thalidomide/  Dexamethasone | Multiple Myeloma | Inhibition of Caspase 1  activation | Phase 3 | NCT00461747 |
| Biological: filgrastim / cisplatin / cyclophosphamide / dexamethasone / etoposide / thalidomide | Multiple Myeloma | Inhibition of Caspase 1  activation | Phase 3 | NCT00005834 |
| Thalidomide / Cyclophosphamide / Velcade / Dexamethasone | Multiple Myeloma | Inhibition of Caspase 1 activation | Phase 3 | NCT01971658 |
| Thalidomide / Biphosphonates | Multiple Myeloma | Inhibition of Caspase 1  activation | Phase 3 | NCT00222053 |
| Other: Laboratory Biomarker  Analysis / Lenalidomide /  Melphalan / Prednisone /Other: Quality-of-Life Assessment /  Thalidomide | Plasma Cell Myeloma | Inhibition of Caspase 1  activation | Phase 3 | NCT00602641 |
| Dexamethasone /Other: Laboratory Biomarker Analysis /  Lenalidomide / Thalidomide | DS Stage I Plasma Cell Myeloma /DS Stage II Plasma Cell Myeloma /DS Stage III Plasma Cell Myeloma | Inhibition of Caspase 1  activation | Phase 3 | NCT00098475 |
| zoledronic acid / dexamethasone / thalidomide / vincristine /  adriamycin | Multiple Myeloma | Inhibition of Caspase 1  activation | Phase 3 | NCT00215943 |
| Velcade-Dexamethasone / Velcade-Thalidomide-  Dexamethasone | Multiple Myeloma | Inhibition of Caspase 1  activation | Phase 3 | NCT00910897 |
| Bortezomib / Dexamethasone / Melphalan / Prednisone /  Thalidomide | Multiple Myeloma | Inhibition of Caspase 1  activation | Phase 3 | NCT00507416 |
| Procedure: Single ASCT with  Thalidomide maintenance | Multiple Myeloma | Inhibition of Caspase 1  activation | Phase 3 | NCT00892346 |
| Velcade / Thalidomide /  Dexamethasone /Procedure:  Peripheral Blood Stem Cell (PBSC) collection /Procedure: First  Autologous Transplantation /  Procedure: Second Autologous Transplantation | Multiple Myeloma | Inhibition of Caspase 1  activation | Phase 3 | NCT01134484 |
| Bortezomib, cyclophosphamide, thalidomide, dexamethasone | Multiple Myeloma /Effects of Chemotherapy | Inhibition of Caspase 1  activation | Phase 3 | NCT03402295 |
| Induction (intensive pathway) –  cyclophosphamide, lenalidomide, & dexamethasone (CRD) regimen / Induction (intensive pathway) –  cyclophosphamide, thalidomide, & dexamethasone (CTD) regimen / Induction (intensive pathway) –  carfilzomib, cyclophosphamide,  lenalidomide, & dexamethasone (CCRD) regimen / Induction  (non-intensive pathway) - cyclophosphamide, lenalidomide, &  dexamethasone attenuated (CRDa) regimen / Induction (non-intensive pathway) - cyclophosphamide,  thalidomide, & dexamethasone  attenuated (CTDa) regimen /  Consolidation (intensive & non-intensive pathways) - bortezomib, cyclophosphamide, & dexamethasone (VCD) regimen / Maintenance  (intensive & non-intensive pathways) - lenalidomide maintenance / Maintenance (intensive & non-intensive pathways - protocol v5.0 only) - lenalidomide plus vorinostat maintenance / High dose  melphalan therapy and autologous stem cell transplant  (intensive pathway only) | Multiple Myeloma | Inhibition of Caspase 1  activation | Phase 3 | NCT01554852 |
| Thalidomide | Multiple Myeloma | Inhibition of Caspase 1  activation | Phase 3 | NCT00367185 |
| Clarithromycin / Thalidomide / Cyclophosphamide /  Dexamethasone | Multiple Myeloma | Inhibition of Caspase 1  activation | Phase 3 | NCT02248428 |
| Thalidomide / Bortezomib /  Cyclophosphamide / Dexamethasone / Prednisolone | Multiple Myeloma | Inhibition of Caspase 1  activation | Phase 3 | NCT01539083 |
| Bortezomib (VELCADE), Thalidomide, and Dexamethasone (VTD) / Bortezomib, Thalidomide, Dexamethasone (VTD) + daratumumab / Daratumumab | Multiple Myeloma | Inhibition of Caspase 1  activation | Phase 3 | NCT02541383 |
| Bortezomib,Pirarubicin,Dexamethasone / Thalidomide,  Pirarubicin,Dexamethasone | Multiple Myeloma | Inhibition of Caspase 1  activation | Phase 4 | NCT01249690 |
| Interim/Maintenance  Dexamethasone / Induction/  Consolidation Dexamethasone /  Induction/Consolidation Cisplatin / Induction/Consolidation  Adriamycin / Induction  Consolidation Cyclophosphamide / Induction/Consolidation Etoposide / Induction Pegfilgrastim /  Transplant 1 Dexamethasone / Transplant 1 Cisplatin / Transplant 1 Adriamycin / Transplant 1  Cyclophosphamide / Transplant 1 Etoposide / Transplant 1  Melphalan / Transplant 1 and 2 Pegfilgrastim /Procedure:  Autologous Peripheral Blood Stem Cell Transplant (ASCT) /  Transplant 2 Carmustine /  Transplant 2 Etoposide /  Transplant 2 Cytarabine /  Transplant 2 Melphalan /  Transplant 2 Dexamethasone / Transplant 1 and 2 Bortezomib / Transplant 1 and 2 Thalidomide | Multiple Myeloma | Inhibition of Caspase 1  activation | Phase 3 | NCT00574080 |
| Thalidomide | Multiple Myeloma | Inhibition of Caspase 1  activation | Phase 2 /Phase 3 | NCT00657488 |
| Melphalan/Prednisone/Velcade / Thalidomide/Prednisone/Velcade | Multiple Myeloma | Inhibition of Caspase 1  activation | Phase 3 | NCT00443235 |
| Thalidomide / Dexamethasone | Multiple Myeloma | Inhibition of Caspase 1  activation | Phase 2 /Phase 3 | NCT00038090 |
| Velcade (Bortezomib) / Thalidomide / Dexamethasone | Multiple Myeloma | Inhibition of Caspase 1  activation | Phase 3 | NCT00256776 |
| Thalidomide | Multiple Myeloma | Inhibition of Caspase 1  activation | Phase 3 | NCT00083876 |
| Thalidomide/Dexamethasone vs Melphalan/Prednisone | Multiple Myeloma | Inhibition of Caspase 1  activation | Phase 2 /Phase 3 | NCT00205751 |
| Ixazomib, thalidomide, &  dexamethasone (ITD) re-induction / Conventional autologous stem cell transplant (ASCT-con) /  Augmented autologous stem cell transplant (ASCT-aug) / ITD  consolidation and ixazomib  maintenance vs. No further therapy | Multiple Myeloma | Inhibition of Caspase 1  activation | Phase 3 | NCT03562169 |
| Thalidomide | Waldenstrom Macroglobulinemia | Inhibition of Caspase 1  activation | Phase 4 | NCT02844309 |
| Thalidomide(Thado) | Carcinoma, Hepatocellular | Inhibition of Caspase 1  activation | Phase 3 | NCT00225290 |
| Thalidomide combined with R-CHOP / R-CHOP | Thalidomide | Inhibition of Caspase 1  activation | Phase 3 | NCT03318835 |
| Thalidomide /Other: Observation | Lymphoma, Large B-Cell, Diffuse | Inhibition of Caspase 1  activation | Phase 3 | NCT03016000 |
| Velcade, Thalidomide,  Dexamethasone | Multiple Myeloma | Inhibition of Caspase 1  activation | Phase 3 | NCT00111748 |
| Thalidomide / Cyclophosphamide | Immunoglobulin Light-Chain Amyloidosis | Inhibition of Caspase 1  activation | Phase 4 | NCT04612582 |
| pegaspargase / Gemcitabine / Oxaliplatin / Methotrexate /  Dexamethasone / Thalidomide | Extranodal NK-T-Cell Lymphoma, Nasal and Nasal-Type | Inhibition of Caspase 1  activation | Phase 3 | NCT02085655 |
| thalidomide + prednisone +  methotrexate | T-LGL Leukemia /Clpd-Nk | Inhibition of Caspase 1  activation | Phase 2 /Phase 3 | NCT04453345 |
| Velcade / Thalidomide /  Dexamethasone / Adriamycin / Cisplatin / Cyclophosphamide / Etoposide | Multiple Myeloma | Inhibition of Caspase 1  activation | Phase 3 | NCT00572169 |
